# Prevalence of Temporomandibular Disorder Symptoms Among Dental Students at the Faculty of Dental Medicine in Iași: A Self-Reported Study Based on DC/TMD Criteria

**DOI:** 10.3390/diagnostics15151908

**Published:** 2025-07-30

**Authors:** Eugenia Larisa Tarevici, Oana Tanculescu, Alina Mihaela Apostu, Sorina Mihaela Solomon, Alice-Teodora Rotaru-Costin, Adrian Doloca, Petronela Bodnar, Vlad Stefan Proca, Alice-Arina Ciocan-Pendefunda, Monica Tatarciuc, Valeriu Fala, Marina Cristina Iuliana Iordache

**Affiliations:** 1 Department of Odontology-Periodontology and Fixed Prosthodontics, Faculty of Dental Medicine, Grigore T. Popa University of Medicine and Pharmacy Iasi, 16 Universitatii Street, 700115 Iasi, Romania; eugenia-tarevici@umfiasi.ro (E.L.T.); sorina.solomon@umfiasi.ro (S.M.S.); alice-teodora.rotaru@umfiasi.ro (A.-T.R.-C.); petronela_bodnar@umfiasi.ro (P.B.); vlad-stefan-proca@umfiasi.ro (V.S.P.); alice.ciocan@umfiasi.ro (A.-A.C.-P.); 2 Department of Preventive Medicine and Interdisciplinarity, Faculty of Medicine, Grigore T. Popa University of Medicine and Pharmacy Iasi, 16 Universitatii Street, 700115 Iasi, Romania; adrian.doloca@umfiasi.ro; 3 Department of Implantology, Removable Dentures and Dental Technology, Faculty of Dental Medicine, Grigore T. Popa University of Medicine and Pharmacy Iasi, 16 Universitatii Street, 700115 Iasi, Romania; monica.tatarciuc@umfiasi.ro (M.T.); marina.iordache@umfiasi.ro (M.C.I.I.); 4 Department of Therapeutic Dentistry, Faculty of Dentistry, Nicolae Testemiţanu State University of Medicine and Pharmacy of Chişinău, 165 Ştefan cel Mare și Sfânt Blvd., MD-2012 Chişinău, Moldova; valeriu.fala@usmf.md

**Keywords:** temporomandibular disorders prevalence, dental students, DC/TMD, psychosocial factors

## Abstract

Temporomandibular disorders (TMDs) encompass a heterogeneous group of musculoskeletal and neuromuscular conditions affecting the temporomandibular joint (TMJ) and masticatory system. Due to academic stress and parafunctional habits, dental students may be particularly vulnerable to TMD. **Objective:** To determine the prevalence of TMD symptoms and their psychosocial and functional correlates among students at the Faculty of Dental Medicine, UMPh Iasi, Romania, using the diagnostic criteria for TMD (DC/TMD) self-report axis and axis II instruments. **Methods:** In this cross-sectional survey, 356 volunteer students (66.0% female; mean age, 22.9 ± 3.6 years) out of a total population of 1874 completed an online DC/TMD–based questionnaire. Axis I assessed orofacial pain, joint noises, and mandibular locking. Axis II instruments included the Graded Chronic Pain Scale (GCPS), Jaw Functional Limitation Scale (JFLS-20), Patient Health Questionnaire (PHQ-9), Generalized Anxiety Disorder-7 (GAD-7), and Oral Behaviors Checklist (OBC). Descriptive statistics summarized frequencies, means, and standard deviations; χ^2^ tests and *t*-tests compared subgroups by sex; Pearson correlations explored relationships among continuous measures (α = 0.05). **Results:** A total of 5% of respondents reported orofacial pain in the past 30 days; 41.6% observed TMJ noises; 19.7% experienced locking episodes. Mean JFLS score was 28.3 ± 30.5, with 4.8% scoring > 80 (severe limitation). Mean PHQ-9 was 5.96 ± 5.37 (mild depression); 15.5% scored ≥ 10. Mean GAD-7 was 5.20 ± 4.95 (mild anxiety); 16.0% scored ≥ 10. Mean OBC score was 12.3 ± 8.5; 30.1% scored ≥ 16, indicating frequent parafunctional habits. Symptom prevalence was similar by sex, except temporal headache (43.4% females vs. 24.3% males; *p* = 0.0008). Females reported higher mean scores for pain intensity (2.09 vs. 1.55; *p* = 0.0013), JFLS (32.5 vs. 18.0; *p* < 0.001), PHQ-9 (6.43 vs. 5.16; *p* = 0.048), and OBC (13.9 vs. 9.7; *p* = 0.0014). Strong correlation was observed between PHQ-9 and GAD-7 (r = 0.74; *p* < 0.001); moderate correlations were observed between pain intensity and PHQ-9 (r = 0.31) or GAD-7 (r = 0.30), between JFLS and pain intensity (r = 0.33), and between OBC and PHQ-9 (r = 0.39) (all *p* < 0.001). **Conclusions:** Nearly half of dental students reported TMD symptoms, with appreciable functional limitation and psychosocial impact. Parafunctional behaviors and psychological distress were significantly associated with pain and dysfunction. These findings underscore the need for early screening, stress-management interventions, and interdisciplinary care strategies in the dental student population.

## 1. Introduction

Temporomandibular disorder (TMD) refers to a group of over 30 conditions of musculoskeletal and neuromuscular origin that significantly affect the function of the masticatory system and are defined by characteristic signs and symptoms such as pain, joint sounds, and mandibular dysfunction [1,2,3,4,5,6]. These disorders have a substantial impact on quality of life and continue to pose diagnostic and therapeutic challenges despite extensive clinical research [1,2]. TMD involves the temporomandibular joints (TMJs), masticatory muscles, and adjacent tissues and has a high prevalence in the general population [3,4,5,6].

The reported prevalence of TMD varies depending on the studied population and the diagnostic criteria applied [7,8,9,10]. Epidemiological studies indicate that approximately 60–70% of individuals experience at least one TMD symptom during their lifetime, but only 5–12% develop clinical forms requiring treatment [11]. The condition affects up to 15% of adults, with peak incidence between 20 and 40 years of age [6,11,12], and is considered the second most common musculoskeletal condition after chronic low back pain, as well as the most common type of non-odontogenic orofacial pain [1,13]. Epidemiological studies report prevalence rates ranging from ~7% to 30% [14,15], and these rates can reach up to 77% in certain subgroups [16]. TMD is more commonly reported in females, with a ~1.5–2 times higher risk than males [16,17,18,19].

Common symptoms include orofacial pain, joint noises, limited mandibular movements, headaches, and earaches [6,12,20]. Severe manifestations, such as persistent pain, joint locking, or pronounced masticatory dysfunction, can interfere with essential functions like eating and speaking, leading patients to seek care [4,5,14,21]. In such cases, early recognition and management of contributing factors are critical for improving prognosis and reducing long-term impact.

TMD is frequently associated with a broad spectrum of comorbid conditions, which can be categorized as either painful or non-painful, underscoring its multifactorial and systemic nature. Painful comorbidities commonly include fibromyalgia, chronic tension-type headache, migraine, chronic low back pain, vulvodynia, temporomandibular arthralgia, myalgia, interstitial cystitis, and endometriosis. These overlapping chronic pain syndromes may share common neurobiological mechanisms and can amplify the perceived burden of TMD symptoms [22,23]. Non-painful comorbidities may involve sleep disorders, chronic fatigue syndrome, irritable bowel syndrome, tinnitus, gastroesophageal reflux disease, depression, anxiety, post-traumatic stress disorder, and multiple chemical sensitivity. These conditions may influence pain perception, emotional regulation, and general health-related quality of life, further complicating diagnosis and management [22,23,24].

The etiology of TMD is multifactorial involving biological, anatomical, psychosocial, and behavioral components [6,12]. Factors such as trauma, parafunctional habits (bruxism, clenching), and psychological stress (anxiety, depression) have all been implicated in the onset or aggravation of symptoms [5,25,26]. In particular, anxiety and stress, may exacerbate parafunctions and modify pain perception, leading to increased joint and muscle overload [27,28].

Diagnosis is often based on patient history and clinical examination [29]. The Diagnostic Criteria for Temporomandibular Disorders (DC/TMD), developed in 2014, represent the current gold standard for TMD assessment, providing a validated and widely adopted framework for both clinical and research contexts [17,22,27,29]. This validated and widely adopted system uses a dual-axis model: axis I assesses physical symptoms, while axis II evaluates psychosocial status and functional impact. Instruments such as the Graded Chronic Pain Scale (GCPS), Jaw Functional Limitation Scale (JFLS), PHQ-9, GAD-7, and the Oral Behavior Checklist (OBC) allow for multidimensional evaluation of symptom severity, functional limitation, and psychological distress [17,22,27,29,30,31].

Accurate diagnosis and management of TMD often require an integrated, multidisciplinary approach involving general medical assessment, psycho-emotional screening, clinical examination of the masticatory system, and when indicated, imaging techniques such as CBCT or MRI [6,12,22,32,33]. However, the DC/TMD guidelines stress that formal diagnosis should be based on a combination of symptoms and clinical findings. Joint sounds or radiological signs alone, in the absence of pain or functional limitation, are insufficient for diagnosis [22]. This distinction is essential for avoiding overdiagnosis and ensuring a patient-centered treatment strategy,

Used appropriately, the DC/TMD criteria provide clinicians with a standardized, cost-effective framework for the precise diagnosis and management of TMD, improving both care quality and health outcomes.

Dental students represent a population exposed to significant academic pressure and psychosocial stress, especially during clinical training years [34,35,36]. The literature suggests that such stressors may increase their risk of developing TMD symptoms [30,31,34]. Assessing the prevalence and profile of TMD in this group is therefore relevant for designing preventive or support interventions, including stress management strategies.

The present study aims to evaluate, using the DC/TMD questionnaire, the prevalence and characteristics of self-reported temporomandibular disorder (TMD) symptoms among dental students at the Faculty of Dental Medicine in Iași. It also explores associations with psychosocial indicators, gender differences, and functional limitations in order to better understand symptom burden and to inform future support and prevention strategies within this academic population.

## 2. Materials and Methods

### 2.1. Study Design and Participants

We conducted an analytical cross-sectional study on 356 students from the Faculty of Dental Medicine in Iași, Romania. Data were collected using a standardized DC/TMD questionnaire (Romanian, French, and English versions) distributed via the Google Forms platform (Google LLC, Mountain View, CA, USA) between November 15 and December 15. Participants were voluntarily recruited from all years of study (I–VI), with ages mostly ranging between 18 and 26 years (mean age: ~22 years).

**Figure 1:**
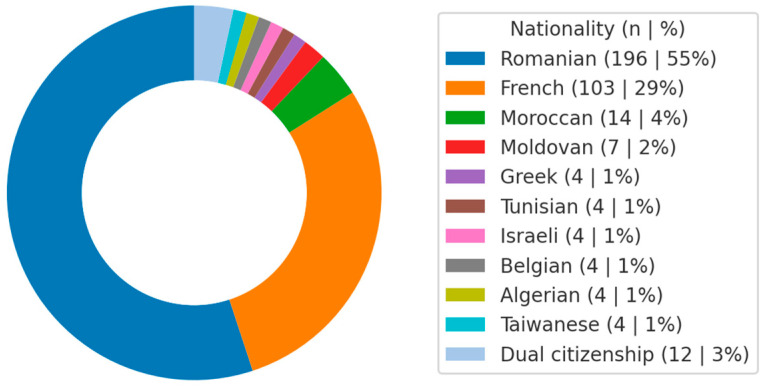
Distribution of study group subjects according to citizenship.

The invitation to participate in the questionnaire study was distributed online via the WhatsApp platform to year leaders, who then forwarded it to all their colleagues (*n* = 1874) across the 6 years of study and the three study tracks—Romanian, French, and English [37]. Among respondents, 55% identified as being of Romanian ethnicity and nationality (the rest came from other cultural backgrounds or were international students) (Figure 1).

The representative sample size for the total number of students (*n* = 1874) was calculated for a 95% confidence level (z = 1.96) and a margin of error of ±5%. The result indicated that a minimum sample of 319 students was required [38]. The questionnaire was completed by 356 students, representing approximately 19% of the total students in the targeted clinical years. Participant selection was voluntary, and no rewards or incentives were offered. All respondents completed the questionnaire in full, making the collected data valid and usable, and no response sets had to be excluded. Since the questionnaire was completed voluntarily and anonymously, the possibility of selection bias cannot be excluded, as students experiencing specific TMD symptoms may have been more likely to participate. Inclusion criteria were active student status at the mentioned faculty and the provision of informed consent to participate, as indicated by completing the questionnaire. It was self-administered under conditions ensuring complete anonymity.

The study was conducted in accordance with the ethical principles of the Declaration of Helsinki, and the research protocol was approved by the University Ethics Committee (approval number: 424/3 April 2024).

### 2.2. Instruments and Measured Variables

The applied questionnaire incorporated DC/TMD diagnostic criteria on axis I (symptomatic part) and several axis II assessments to evaluate impact and psychosocial factors. Specifically, the instrument included the following:

- TMD screening questions (axis I)—the presence of orofacial pain (in the jaw, temple, or ear region) in the last 30 days, pain characteristics (intermittent vs. constant), duration of symptoms (in months), laterality (unilateral/bilateral), the presence of jaw pain/stiffness upon waking, presence of pain triggered or worsened by specific activities (chewing hard foods, yawning/wide mouth opening, teeth clenching or grinding, prolonged speaking, kissing), the presence of joint noises (TMJ clicking or popping), episodes of mandibular joint locking on opening or closing (temporary inability to open the mouth fully, or open-locking—“subluxation”), interference of such locking with eating, headaches in the temporal region associated with mandibular movements. Responses were recorded as yes/no or on nominal scales describing the presence/absence of pain.
- Graded Chronic Pain Scale (GCPS, axis II)—this instrument assessed the intensity and degree of functional impairment due to facial pain. On a scale from 0 to 10, students rated current facial pain intensity (at the time of completion), the most intense pain in the past 30 days, and average pain intensity over the past 30 days. They also reported the number of days (in the past 6 months) with facial pain and the number of days in the past month when pain interfered with normal activities (work, school, and household tasks). Pain interference with daily, social/family, and work activities was rated on a scale from 0 (“not at all”) to 10 (“extremely”). These data allow the classification of subjects by chronic pain grade (0—no pain, I—mild pain, II—moderate pain, III/IV—severe pain with disability).
- Jaw Functional Limitation Scale (JFLS-20, axis II)—used to evaluate the impact of TMD on jaw function. The JFLS contains 20 items assessing difficulty level (0 = no difficulty, 10 = impossible) in performing various jaw-related activities. Questions cover masticatory functions (chewing foods of different consistencies, biting into foods), speech and facial expression functions (opening the mouth for speaking, yawning, singing, smiling, facial expressions), and general oral functions (swallowing, kissing). The total JFLS score (0–100) is calculated as the mean of all item scores × 10, with higher values indicating more severe functional limitation.
- Patient Health Questionnaire (PHQ-9)—for depressive symptoms. This standardized tool asks respondents to assess the frequency—on a scale from 0 (“not at all”) to 3 (“nearly every day”) —of 9 depression symptoms (low interest, sad mood, sleep disturbances, fatigue, appetite changes, feelings of worthlessness or guilt, concentration problems, psychomotor slowing/agitation, thoughts of death) over the past 2 weeks. The total PHQ-9 score (0–27) reflects depression severity (0–4, minimal; 5–9, mild; 10–14, moderate; ≥15, moderate–severe or severe). An additional question asked how difficult these problems made daily life (response options: “Not difficult at all,” “Somewhat difficult,” “Very difficult,” “Extremely difficult”).
- Generalized Anxiety Disorder Questionnaire (GAD-7)—for anxiety symptoms. Like the PHQ-9, the GAD-7 assesses seven generalized anxiety symptoms (nervousness, inability to control worry, excessive worrying, difficulty relaxing, restlessness, irritability, fear of impending doom) on a 0–3 scale over the past 2 weeks. The total score (0–21) indicates anxiety severity (0–4, minimal; 5–9, mild; 10–14, moderate; ≥15, severe). At the end, participants also indicated how much these problems interfered with daily functioning (“Not difficult at all” to “Extremely difficult”).
- Oral Behaviors Checklist (OBC)—a list of 18 oral parafunctional behaviors, including two related to sleep (nocturnal bruxism—teeth grinding or clenching during sleep; sleeping in positions that apply pressure to the jaw) and six related to waking hours (teeth grinding/clenching during the day; frequent tooth contact; jaw muscle tension without tooth contact; pushing or shifting the jaw to one side, etc.). Respondents rated the frequency of each habit on a scale from 0 (“never”) to 4 (“always”). A total OBC score was calculated by summing the scores of 8 key behaviors (maximum theoretical score: 32), with higher scores indicating more frequent or multiple parafunctions.

### 2.3. Statistical Analysis

The collected data were entered and verified in Microsoft Excel and then analyzed using IBM SPSS Statistics v.26. Descriptive analysis was performed for all investigated parameters. Categorical variables (e.g., symptom presence, yes/no answers) were summarized as frequencies and percentages, while continuous variables were reported as mean and standard deviation (or median and interquartile range, depending on distribution).

To assess associations between categorical variables, chi-square (χ^2^) tests of independence were used, with a significance threshold of *p* < 0.05. One such analysis evaluated differences in TMD symptom prevalence by sex (female vs. male).

Associations between the presence of certain parafunctional habits (e.g., bruxism) and symptoms (pain, joint noises) were also tested. For continuous variables, subgroup comparisons (e.g., sex differences in PHQ-9, GAD-7, JFLS scores) were conducted using Student’s *t*-tests (for approximately normal data) or Mann–Whitney nonparametric tests (if distributions were non-normal) with adjustments for unequal variances where necessary.

Correlations between continuous clinical variables (pain intensity, questionnaire scores) were evaluated using Pearson’s correlation coefficient (r) or Spearman’s (ρ) if the data were ordinal. Particular attention was given to correlations between facial pain intensity and psychosocial scores (depression, anxiety) and between jaw dysfunction score (JFLS) and other clinical parameters.

The analysis was performed in line with initial hypotheses, focusing on identifying the prevalence of TMD and significant relationships between variables. The standardized scoring form used in the DC/TMD diagnostic system to interpret questionnaire and clinical evaluation results is a visual tool for classifying symptoms and dysfunction severity based on the scores obtained by the patient.

## 3. Results

### 3.1. Sample Characteristics

**Figure 2:**
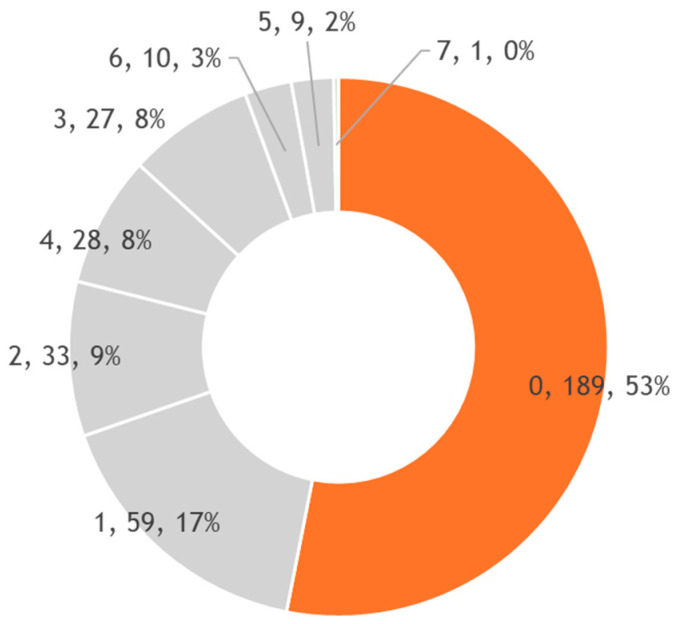
Distribution of TMD Screening Questionnaire scores.

A total of 356 students completed the questionnaire, of whom 235 (66.0%) were female and 115 (32.3%) were male (6 respondents either preferred not to disclose their gender or identified as non-binary). The mean age of participants was 22.9 ± 3.6 years, ranging from 18 to 42 years (with the vast majority—over 95%—being in their 20 s and 30 s, corresponding to typical university age). Among the respondents, 94.7% identified as being of Romanian ethnicity and nationality (the remainder came from other cultural backgrounds or were international students). The distribution of scores for the TMD Screening Questionnaire is presented in the following chart (Figure 2). Across the entire sample, 248 participants (70%) were completely negative. A total of 30% of the sample (108 participants) were entirely positive.

Participants with a score < 2 (screening negative):

- Score 0: 189 individuals (53%)
- Score 1: 59 individuals (17%)

Participants with a score ≥ 2 (considered screening positive):

- Score 2: 33 (9%)
- Score 3: 27 (8%)
- Score 4: 28 (8%)
- Score 5: 9 (2%)
- Score 6: 10 (3%)
- Score 7: 1 (0%)

### 3.2. Prevalence of TMD Symptoms

According to the DC/TMD screening criteria (past 30 days), 155 students (43.5%) reported orofacial pain in the jaw, temple, and/or preauricular region (in front of the ear), either unilaterally or bilaterally. In the vast majority of cases, this pain was intermittent—“the pain comes and goes”—with approximately 97% of those reporting pain describing it this way. Only three students (0.8% of the total sample) reported continuous and constant pain over the past month. The median duration of TMD pain among symptomatic individuals was approximately 4 months (IQR ~2–12 months), indicating that many students were experiencing relatively recent symptoms.

**Figure 3:**
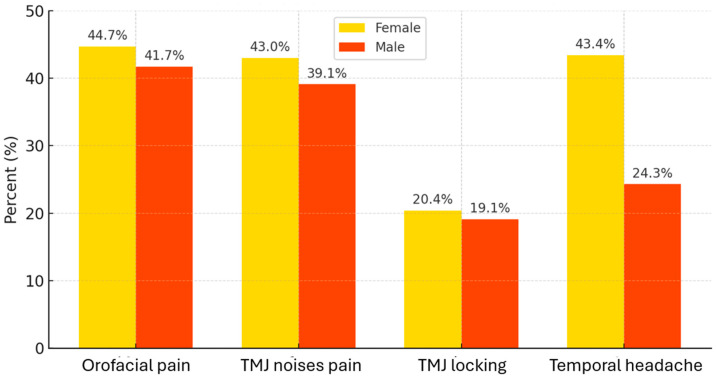
Prevalence of TMD symptoms among students (females vs. males).

Figure 3 illustrates the prevalence of the main reported TMD symptoms compared by sex. Approximately similar proportions of female and male students reported orofacial pain (44.7% vs. 41.7%) or TMJ noises (43.0% vs. 39.1%).

A significant proportion of participants reported joint noises in the temporomandibular joint (TMJ)—148 students (41.6%) noticed clicking or joint sounds during mandibular movements.

Regarding joint-locking phenomena, 70 students (19.7%) experienced one or more episodes of jaw “locking” in the closed position (temporary inability to open the mouth fully). Of these, 36 students (10.1% of the total) indicated that the locking limited their mouth opening to the extent that it affected their eating ability, suggesting episodes of severe locking indicative of possible disc displacement without reduction. Another 33 students (9.3%) described episodes in which the jaw “does not open fully, but then releases,” characteristic of intermittent locking with reduction (the articular disc temporarily interferes with movement and then spontaneously returns to position).

At the time of evaluation, only 12 participants (3.4%) reported that their jaw currently felt locked or restricted in movement on a regular basis (persistent). As for open-locking episodes (mandibular hypermobility), 28 students (7.9%) had at least one episode of jaw locking when yawning or opening their mouth very wide, and 25 students (7.0%) reported having to “do something to close the mouth” after such an episode, indicating more pronounced mandibular subluxation/hypermobility.

The average duration of TMD-related pain in symptomatic individuals was approximately 4 months (IQR ~2–12 months), suggesting that many students were experiencing relatively recent symptoms.

Approximately similar proportions of female and male students reported orofacial pain (44.7% vs. 41.7%) or TMJ noises (43.0% vs. 39.1%) (Figure 3).

In addition to pain localized in the TMJ region, 131 students (36.8%) also reported headaches in the temporal area (temples), which, in the context of DC/TMD, may be associated with pain in the temporalis muscle (headache attributed to TMD). Among those with temporal headaches, approximately 25% noticed that it occurs or worsens when chewing hard foods, 30% noticed that it occurs or worsens during teeth clenching, and 22% noticed that it occurs or worsens when opening the mouth wide, suggesting a functional link between mandibular activity and headache.

### 3.3. Detailed Descriptive Analysis of Symptoms

**Figure 4:**
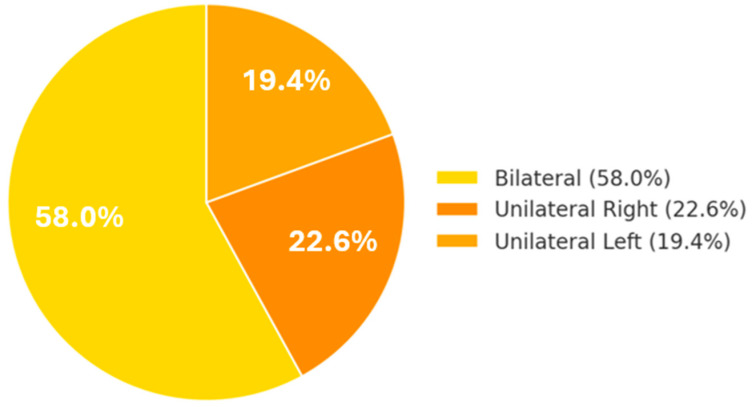
Location of oro-facial pain (*n* = 155).

Among students with orofacial pain (*n* = 155), the characteristics were as follows: the pain was bilateral in 58.1% of cases and unilateral in 41.9% (with no marked lateral preference—22.6% on the right side, 19.4% on the left) (Figure 4).

**Figure 5:**
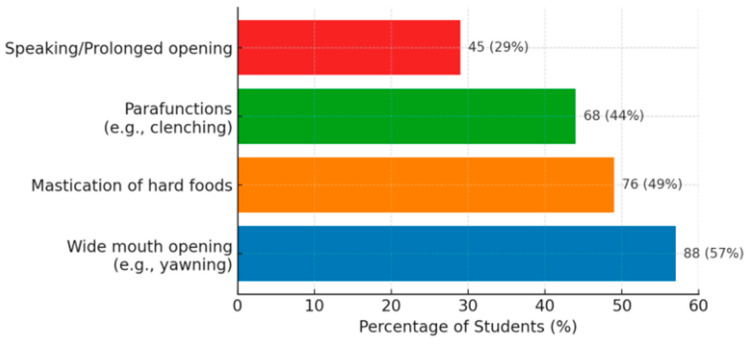
Major aggravating factors of pain (*n* = 155).

In 68 cases (19.1% of the total sample), students reported jaw pain or stiffness upon waking in the morning. This morning symptom (often considered an indicator of nocturnal bruxism) was predominantly reported by those who also experienced pain during the day: contingency analysis showed that the presence of morning pain/stiffness was significantly associated with general orofacial pain (χ^2^ with 1 df = 34.1, *p* < 0.001). Regarding the triggering or aggravating factors of mandibular pain, the most frequently reported were wide mouth opening (e.g., yawning, biting into large foods)—mentioned by 74 students (20.8%); parafunctional habits (e.g., clenching or grinding teeth, chewing gum)—mentioned by 69 students (19.4%); chewing hard or sticky foods—reported by 61 students (17.1%); and activities involving prolonged speaking, singing, or yawning—reported by 42 students (11.8%). These proportions are calculated relative to the entire sample; when considered only among subjects with pain, approximately half to two-thirds experience pain aggravation during such functional activities. Thus, chewing worsens the pain in ~49% of those with pain, extreme mouth opening worsens the pain in ~57%, parafunctional habits (clenching) worsen the pain in ~44%, and speaking/yawning worsens the pain in ~29%. These data highlight the musculoskeletal nature of the pain—activities that strain the muscles and joints cause discomfort, characteristic of TMD (Figure 5).

#### 3.3.1. Facial Pain Intensity and Impact

On a 0–10 scale, the mean reported facial pain intensity (GCP item 4.4) was 1.90 ± 2.49. Approximately 45% of students reported at least mild pain at the time of completing the questionnaire (score > 0 for “pain now”), but for most, the intensity was low (median = 0, with first quartile = 0 and third quartile = 3). The highest pain experienced in the past month had a mean intensity of 2.20 ± 2.91, with 16.6% of students reporting moderately or severely painful episodes (score ≥ 5).

**Figure 6:**
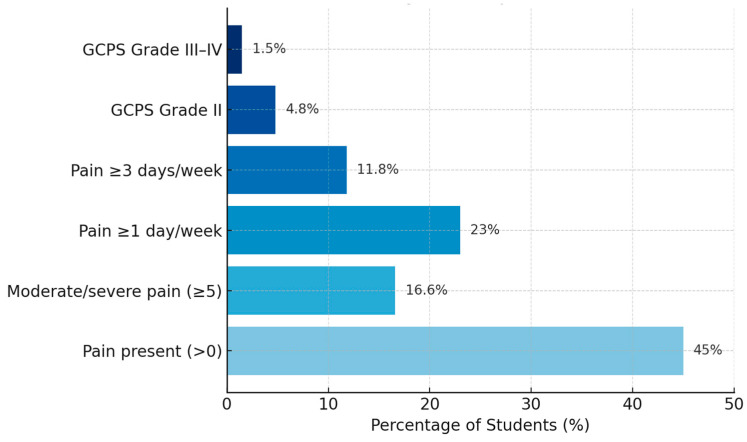
Facial pain intensity and impact indicators (*n* = 356).

Regarding chronic pain, 23.0% of the sample reported pain occurring ≥1 day per week over the past 6 months (≥24 days in 6 months), and 11.8% reported pain on ≥3 days per week (≥72 days in 6 months). According to the GCPS classification, most students fell into grade 0 (no significant pain) or grade I (mild pain, no disability); only 4.8% were classified as grade II (moderate pain), and less than 2% as grades III–IV (severe pain with functional impact) (Figure 6).

The mean number of activity-affected days due to pain (in the past month) was very low (mean ~1 day; median = 0, IQR 0–0), confirming that for the majority of students, the pain—although present—did not significantly interfere with daily activities. However, a small subgroup (~5%) reported that facial pain interfered with daily tasks for more than 7 days in the past month.

#### 3.3.2. Mandibular Functional Limitation Score (JFLS)

**Figure 7:**
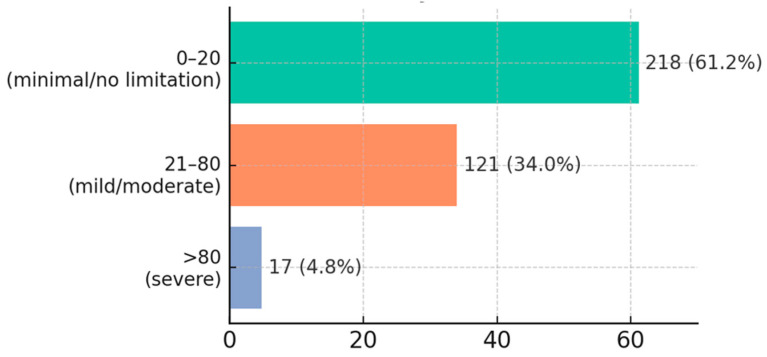
Distribution of JFLS score.

The mean JFLS score (0–100) across the entire sample was 28.3 ± 30.5 (Figure 7). This relatively low average masks significant variability: most students without pain or with minimal symptoms had very low scores (0–20), indicating no or minimal functional limitations (e.g., slight difficulty chewing tough foods). In contrast, 17 students (~4.8%) had scores above 80, suggesting severe functional limitations (major difficulty performing many mandibular activities).

The most affected functions (on average) were chewing hard foods (mean score of 3.1/10 at group level, with 15% of students reporting a difficulty of ≥5/10 for this item) and yawning/maximal mouth opening (12% reported moderate or severe difficulty).

#### 3.3.3. Psychosocial Scores (Axis II)

**Figure 8:**
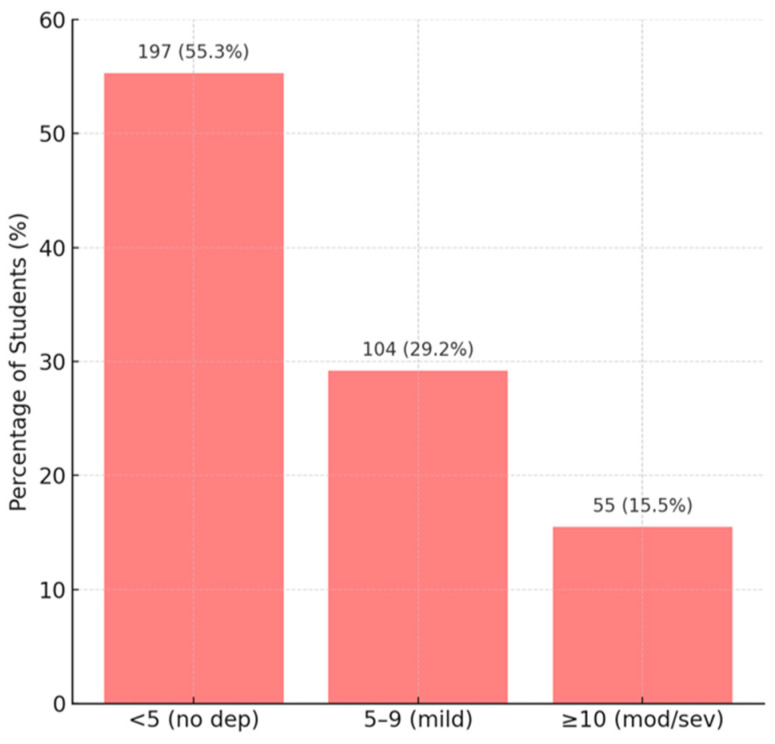
Distribution of PHQ-9 scores (clinical depression).

**Figure 9:**
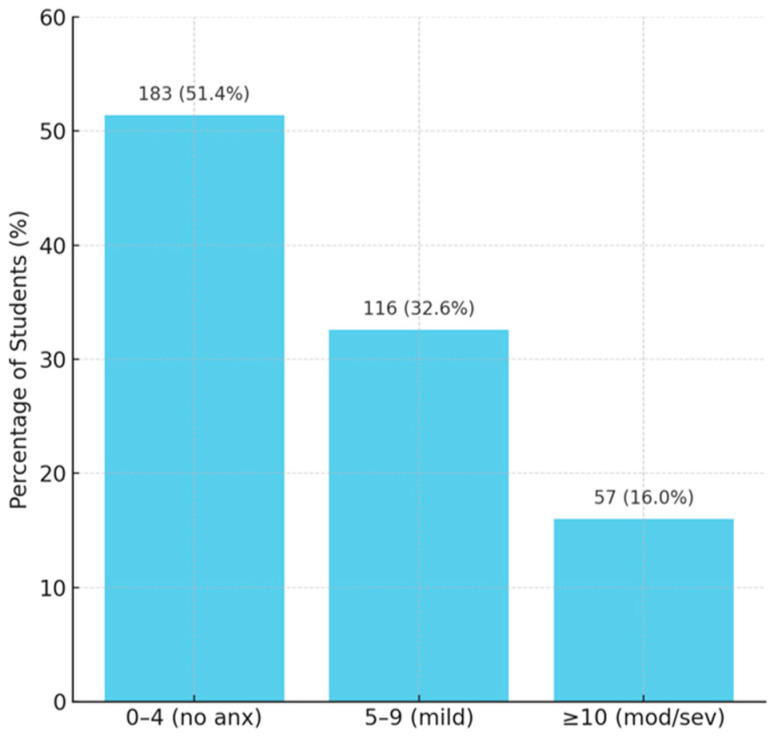
Distribution of GAD-7 scores (anxiety).

The mean PHQ-9 score (depression) was 5.96 ± 5.37, which corresponds, at the group level, to mild depression (5–9) (Figure 8). The distribution showed that 197 students (55.3%) had practically no significant depressive symptoms (score < 5), 104 (29.2%) had mild depression (PHQ between 5–9), and 55 (15.5%) had a score ≥ 10 (possible moderate or severe depression, requiring clinical evaluation). Similarly, for anxiety (GAD-7), the mean score was 5.20 ± 4.95 (indicating mild anxiety on average); 183 students (51.4%) had no notable anxiety (score 0–4), 116 (32.6%) had mild anxiety, and 57 (16.0%) had moderate to severe anxiety (score ≥ 10) (Figure 9). These figures are comparable to known data for the young university population.

Regarding the questions about the difficulty caused by mental health problems, approximately 41% acknowledged “some difficulty” in daily life due to depressive symptoms, while 3% considered them “very difficult” or “extremely difficult.” For anxiety, ~40% reported “some difficulty,” and ~8% rated it as “very/extremely difficult.”

**Figure 10:**
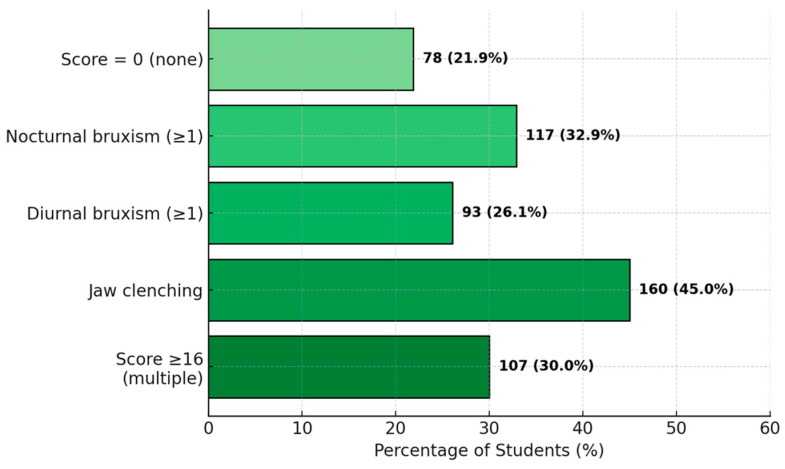
Distribution of parafunctional habits (PH).

The total score for oral parafunctional habits (OBCs) ranged from 0 to 29 (out of a maximum of 32), with a mean of 12.3 ± 8.5. Only 21.9% of students had an OBC score of 0 (meaning they reported never engaging in any of the listed parafunctional habits) (Figure 10). Most students admitted to engaging in at least some parafunctions occasionally: a total of 32.9% reported clenching or grinding their teeth during sleep (nocturnal bruxism) with any frequency (score ≥ 1 on the corresponding item), and 26.1% reported daytime bruxism (teeth grinding during the day, score ≥ 1). However, the most common parafunctional habit was holding the jaw in a clenched position or keeping the masticatory muscles tense without dental contact—approximately 45% admitted to doing this occasionally, likely involuntarily in situations involving concentration or stress. Approximately 30% of students had an OBC score ≥ 16 (indicating multiple and frequent parafunctional habits), suggesting that one-third exhibit behaviors that may predispose them to TMD, such as bruxism.

#### 3.3.4. Associations Between Variables (χ^2^ Tests)

**Figure 11:**
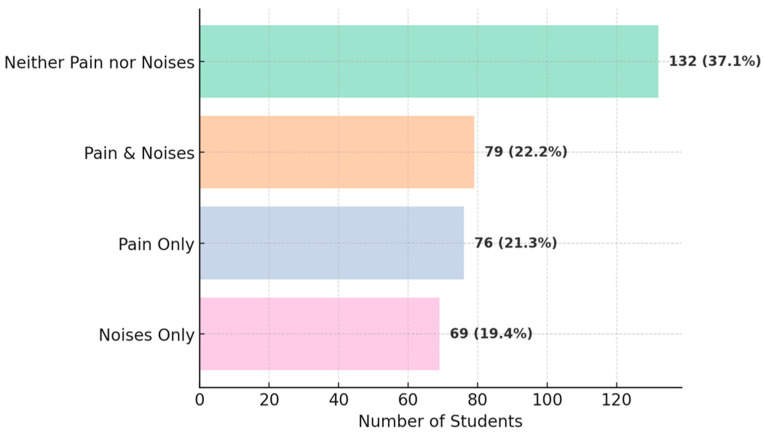
Associations: oro-facial pain vs. TMJ noises.

**Figure 12:**
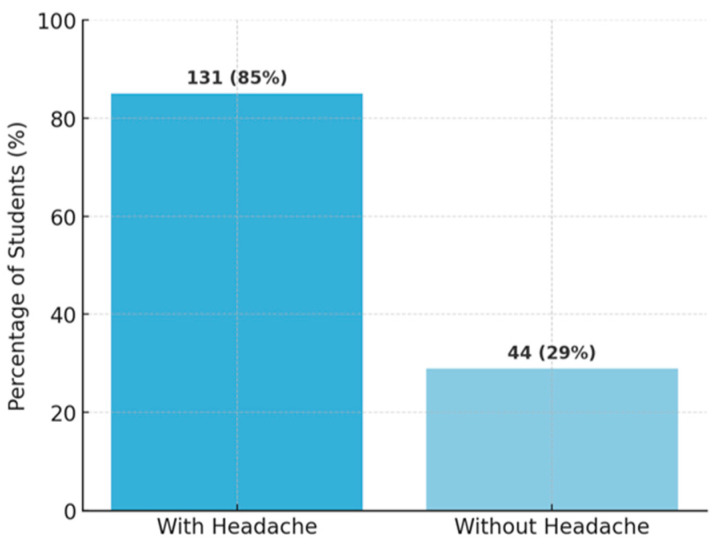
Associations: oro-facial pain and headache presence.

**Figure 13:**
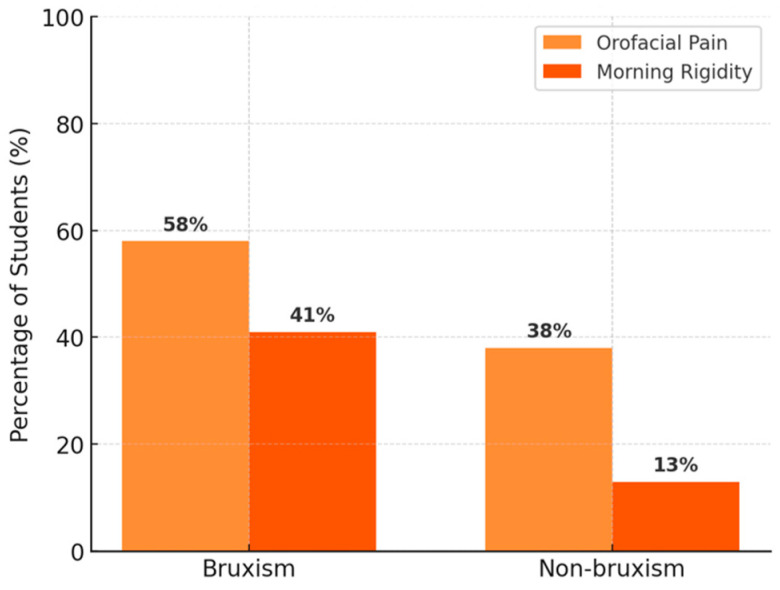
Associations: nocturn bruxism and symptoms.

We investigated the associations between TMD symptoms and demographic or clinical characteristics (Table 1). A notable result was the association between the presence of orofacial pain and TMJ noises: 79 students presented with both pain and noises, 132 had neither pain nor noises, while only a minority had just one type of symptom (76 reported pain without noises, 69 reported noises without pain) (Figure 11). This association is statistically significant (χ^2^ with 1 df = 9.84, *p* ≈ 0.002), suggesting that individuals with pain are more likely to also experience joint noises (and vice versa) compared to asymptomatic subjects. Additionally, temporal headaches were strongly associated with orofacial pain: 85% of those with temporal headache also reported pain in the TMJ region compared to only 29% of those without headache (*p* < 0.001) (Figure 12). A link was also observed between parafunctional habits and symptoms: students who reported nocturnal bruxism (teeth grinding during sleep) had a significantly higher prevalence of orofacial pain (58% vs. 38% in non-bruxers, χ^2^ = 10.8, *p* = 0.001) and morning jaw stiffness (41% vs. 13%, χ^2^ = 32.7, *p* < 0.001) (Figure 13). The presence of TMJ noises was associated with a higher OBC score (mean 15.0 vs. 10.6, *p* < 0.01), mainly due to clenching/grinding-type parafunctions. These findings support the idea of an etiological link between parafunctional habits (which overload the joint) and TMD manifestations.

#### 3.3.5. Differences Between Subgroups (Female vs. Male)

Figure 3 shows that the prevalence of symptoms (pain, joint noises, locking) was comparable between the two genders. Chi-square tests did not indicate significant differences in orofacial pain (44.7% of females vs. 41.7% of males, χ^2^ = 0.17, *p* = 0.68), TMJ noises (43.0% vs. 39.1%, *p* = 0.57), or closed mandibular locking (20.4% vs. 19.1%, *p* = 0.88). In contrast, temporal headache was much more frequent among female students (43.4% vs. 24.3% in male students; χ^2^ = 11.2; *p* = 0.0008) (Table 2). Thus, women appear more prone to the myofascial components (muscle pain in the temple area) of TMD in this sample, even though the frequency of reported joint pain does not differ significantly.

However, the comparative analysis indicated notable differences in symptom severity and their consequences between sexes. Female students recorded, on average, higher scores across several clinical parameters: the mean intensity of facial pain in females was 2.09 vs. 1.55 in males (*t*-test, *p* = 0.0013), and the average number of pain days over six months was nearly double (10.8 vs. 4.9 days, *p* = 0.02). Additionally, the JFLS score was considerably higher in women—with a mean of 32.5 compared to 18.0 in men (*p* < 0.001)—suggesting that women experience more pronounced functional limitation at the mandibular level (although, as mentioned, the rates of reported objective symptoms are similar).

**Figure 14:**
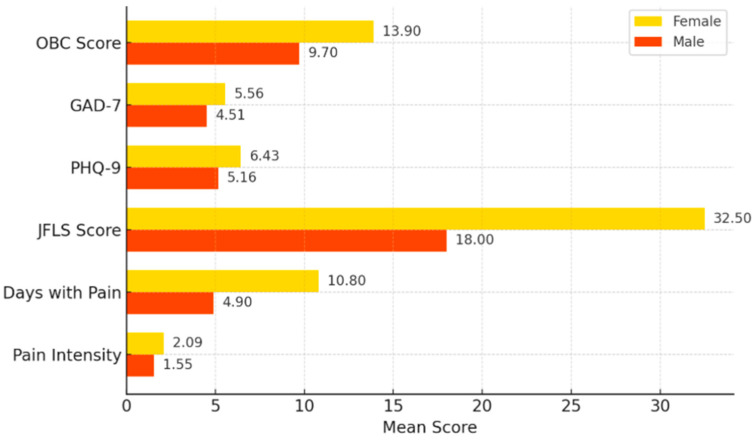
Comparison of mean clinical scores by sex.

At the psychosocial level as well, women had slightly higher scores: a mean PHQ-9 score of 6.43 vs. 5.16 in men (*p* = 0.048), and for GAD-7, a mean of 5.56 vs. 4.51 (*p* = 0.077, not statistically significant but indicative of a trend). The OBC score was also significantly higher in the female group (13.9 vs. 9.7, *p* = 0.0014), indicating a higher frequency of oral parafunctions among female students (Figure 14).

These differences suggest that, although men and women are proportionally equally affected by TMD in terms of basic symptoms, women tend to experience slightly more severe or functionally impactful forms (possibly due to increased vulnerability or more attentive symptom reporting).

#### 3.3.6. Correlations Between Clinical Variables

**Figure 15:**
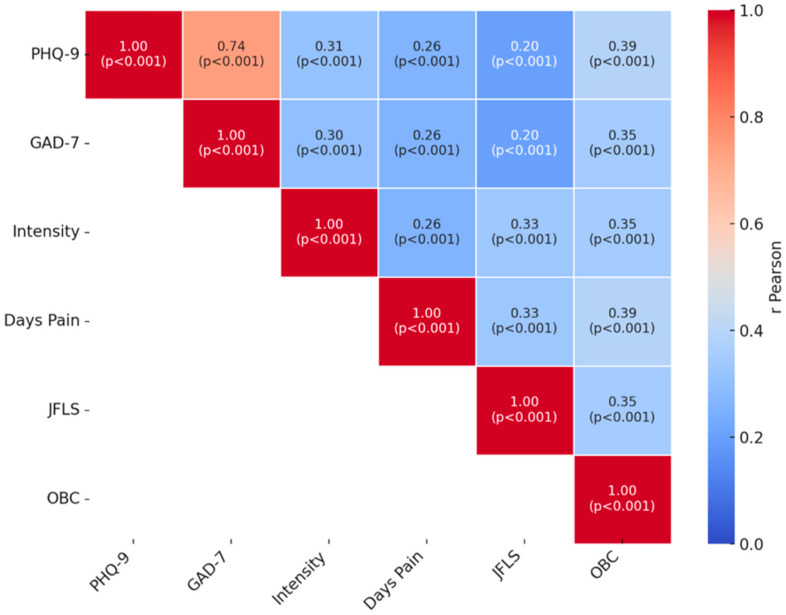
Correlations between clinical variables (r, *p*-values).

The analysis of Pearson correlation coefficients revealed several significant relationships between the continuous measured parameters, supporting the expected theoretical links (Figure 15). First, depression (PHQ-9) and anxiety (GAD-7) scores showed a strong correlation with each other (r = 0.74, *p* < 0.001), indicating significant psycho-emotional comorbidity (students with depressive symptoms also tend to experience anxiety, and vice versa). Both PHQ-9 and GAD-7 scores showed a moderate positive correlation with the average intensity of facial pain (r = 0.31 for PHQ and r = 0.30 for GAD, *p* < 0.001). In other words, students with more intense facial pain tend to report more pronounced emotional symptoms. Similarly, the number of days with pain in the past six months correlated with the PHQ score (r ≈ 0.26), with chronic pain being associated with a more affected depressive status. A significant correlation was also found between the JFLS score (mandibular dysfunction) and the average pain intensity (r = 0.33, *p* < 0.001), confirming that functional limitations increase with the intensity of perceived pain. The JFLS score was also slightly positively correlated with depression/anxiety scores (r~0.20), suggesting that patients with severe joint dysfunctions may also experience psychosocial impact (possibly bidirectional). Another notable aspect was the relationship between parafunctional habits (OBC score) and clinical parameters: the OBC score had a significant positive correlation with both average pain intensity (r = 0.35, *p* < 0.001) and PHQ-9 score (r = 0.39, *p* < 0.001). This indicates that students with more parafunctional habits tend to experience more intense facial pain and also report higher levels of stress/depression. One can imagine a vicious cycle: psycho-emotional stress favors parafunctions (such as jaw clenching in anxious states) [27] which, in turn, overload the TMJ and can generate pain, thus perpetuating the symptomatology.

In summary, the results of our study indicate a high prevalence of TMD symptoms among dental students in Iași—almost half of the respondents reported orofacial pain, and around two-fifths reported joint noises, usually of mild to moderate intensity. Women and men were equally affected by these dysfunctions in terms of frequency, although the subjective impact (pain, dysfunction) was greater in women. Significant associations between psychosocial factors and TMD were confirmed: students with symptoms of anxiety or depression and those with parafunctional habits exhibited TMD manifestations more frequently and with greater intensity.

These results should be interpreted in the context of the data collection method, as self-reporting is susceptible to under- or overestimations depending on the individual perception of symptoms and the level of personal awareness.

## 4. Discussion

The present study provides an up-to-date and comprehensive picture of the prevalence and characteristics of temporomandibular disorders (TMDs) among students at a dental school in Romania using standardized assessment tools (DC/TMD). The contribution of our study—using the current gold-standard DC/TMD instruments and a multidimensional assessment model that includes somatic, functional, and psychological dimensions—lies in the detailed analysis of psychosocial correlates and the functional impact of TMD symptoms in a relatively understudied Eastern European dental student population.

The overall prevalence of TMD symptoms in our sample was substantial—approximately 43.5% of students reported recent orofacial pain, and 64.6% presented at least one TMD sign or symptom (pain, joint noises, and/or mandibular locking). These figures fall within the upper range reported by other similar studies, suggesting that TMJ dysfunctions may be a common issue among young dental students. For example, Srivastava et al. (2021) found a TMD prevalence of 36.99% among dental students in Saudi Arabia [17], while Angeles-García et al. (2025) reported a prevalence of 24.5% among dental students in Peru [27]. Other international investigations indicate similar rates, for instance, ~22% in Thailand [39], ~21% in Jordan [40], and ~16% in Serbia [41] among the general population. Compared to these data, the TMD pain prevalence of ~43.5% among students in Iași is higher.

A possible explanation may lie in methodological differences: our study included self-reported pain episodes during the last month (a highly sensitive measure), which may have captured subclinical or transient cases, whereas other studies used stricter diagnostic criteria and supplemented the assessment with clinical DC/TMD examination (likely excluding isolated noises or minor pain). Additionally, student populations may vary—cultural factors, academic stress levels, and year of study can influence prevalence. Notably, our students came from all academic years, while the Peruvian study analyzed students in their final years who might have been more exposed to clinical stress and the COVID-19 pandemic (yet, paradoxically, showed lower prevalence). These aspects emphasize the need for cautious interpretation of prevalence data, since the elevated rates in our survey likely reflect mostly mild, self-limited, transient symptoms rather than severe disorders. In addition, since participation was voluntary and based on self-reporting, it’s probable that students already experiencing TMD symptoms were more motivated to complete the questionnaire than asymptomatic individuals, which may have slightly inflated the actual prevalence. However, the fact that ~1 in 10 students experienced episodes of severe mandibular locking and ~5% reported moderate-to-severe chronic pain indicates the existence of a non-negligible subgroup that could benefit from evaluation and therapeutic intervention.

The gender distribution of TMD in the literature is classically unbalanced, with women showing a higher susceptibility to chronic orofacial pain. Our study did not find a significant difference in symptom prevalence (44.7% of females vs. 41.7% of males reported pain, *p* = 0.68; TMJ noises, 43.0% vs. 39.1%, *p* = 0.57). This finding is in line with recent data from China on medical students, where TMD prevalence was 31.7%, with no significant gender differences [5]. The Chinese authors hypothesized that similar access to education and living conditions might equalize TMD risk between sexes among young adults. However, the majority of epidemiological studies show a higher risk in women. For example, Srivastava et al. reported that female dental students had an OR = 1.94 (95% CI ~1.24–3.04) for developing TMD compared to their male counterparts [17]. Some differences may arise from case definition criteria: if only clinically confirmed diagnoses are included, it is possible that women (who tend to seek medical care more often for facial pain) are more represented. In our self-reported study, men may have underreported minor symptoms, while women may have overreported, artificially balancing prevalence. Nevertheless, our analyses clearly showed that despite similar prevalence, TMD severity and impact were greater in women—they reported higher pain intensities, more pain days, and significantly higher JFLS scores. This result aligns with clinical observations that women experience chronic pain and associated TMD dysfunction more intensely. Proposed mechanisms include hormonal differences, with estrogens potentially modulating nociceptive processing and increasing joint laxity or inflammatory sensitivity. These factors may explain the greater pain intensity, functional impairment, and frequency of temporal headaches observed among female students despite comparable symptom prevalence. Psychosocial influences may also contribute, as women are more likely to recognize, internalize, and report pain symptoms [42,43,44].

Another gender-related finding in our study was the significantly higher incidence of temporal headaches among women (43% vs. 24%). Tension-type/TMD-related headaches are known to be more prevalent in women in the general population, and our data confirm this pattern. On the other hand, for joint noises and locking—more mechanical phenomena—no notable gender differences were found, suggesting that these manifestations may depend less on gender-related predispositions and more on anatomy or parafunctional habits common to both sexes.

Additionally, recent studies point to the involvement of the trigeminal nociceptive pathways and neuroimmune interactions in TMD-related pain. The activation of glial cells and the release of pro-inflammatory cytokines in the trigeminal system may amplify pain perception and contribute to symptom persistence, particularly in predisposed individuals [26].

An essential finding of the study is the link between psychosocial factors and TMD. Correlation analysis showed that students with high anxiety and depression scores tended to report more intense and more frequent facial pain. This result is supported by the literature: anxiety has been identified as one of the most frequent factors associated with TMD [19,27], increasing the risk of its occurrence (for example, Srivastava et al. found OR = 1.55 for anxiety among students with TMD) [17]. Mechanistically, anxiety may amplify pain perception (via hypervigilance and reduced nociceptive thresholds) and may favor harmful behaviors such as parafunctions (bruxism, jaw clenching as a stress response) [35,45]. Our observation that OBC scores correlate significantly with both pain and depression suggests that parafunctions are a key link between psychological stress and biomechanical loading of the TMJ. Highly anxious students may experience frequent teeth-grinding episodes, which would explain why they had the highest OBC and TMD scores in our sample. These results confirm the validity of the biopsychosocial model of TMD etiology [14,29,43,46]—not only local physical factors but also general emotional state contribute to TMJ dysfunction manifestation. Consequently, the therapeutic approach to patients (even young students) with TMD should be multidisciplinary, combining pain and joint function management with stress-reduction interventions, awareness training for parafunctional habits, and psychological counseling when necessary. Behavioral and lifestyle interventions, such as mindfulness, yoga, physical activity, and relaxation techniques, have shown promising results in reducing stress-related parafunctions and may help alleviate or even reverse mild TMD symptoms, particularly in young adults [21].

When comparing the specific TMD patterns observed in our students with those reported in other studies, interesting similarities emerge. In the Saudi Arabian study on dental students [17], the most common diagnoses were myalgia (masticatory muscle pain)—consistent with orofacial pain being the most common symptom in our study—and disc displacement with reduction (TMJ clicking) as the most prevalent intra-articular disorder, which also corresponds with our data, where ~41.6% reported joint noises. That study also showed that students with confirmed TMD had significantly higher pain intensity and JFLS scores compared to those without TMD—evidence that resonates with our findings on subgroup differences (e.g., females and students with high anxiety presenting higher pain intensities and JFLS scores). Thus, the present study supports the validity of applying DC/TMD criteria in the Romanian academic context, with results aligned with international trends and confirming that known risk factors (female gender, anxiety, parafunctional behaviors) manifest similarly in this population.

A particular feature of our sample is that all participants were dental students, which may influence both the prevalence and the accuracy of self-reported TMD symptoms. Being familiar with dental medicine, these students may more easily recognize specific symptoms (e.g., realizing that a “click” in the jaw is a sign of TMJ dysfunction) and may be more aware of the importance of reporting them. This may lead to a higher self-reported prevalence compared to the general population due to increased awareness. On the other hand, the intense academic stress to which dental students are exposed, especially in clinical years, may make them more vulnerable to TMD. Students in the final years of our sample may experience an increased incidence of musculoskeletal facial pain due to both stress and clinical activities (clinical work may involve awkward postures and visual and muscular strain, all associated with cervical and orofacial tension). Previous studies have suggested that final-year students may have higher TMD prevalence compared to those in preclinical years [5,27,30,31]. At the same time, our study’s exclusive reliance on self-reported data introduces a limitation: students in the earlier years of training may lack the clinical experience required to correctly interpret certain symptoms such as joint sounds, bruxism, or mandibular locking. This may lead to underreporting in this subgroup and could affect the accuracy of comparisons across academic levels. Nevertheless, even under these conditions, the overall prevalence of TMD-related symptoms in our sample remains high, underscoring the importance of this issue among dental students and the need for targeted awareness, screening, and prevention strategies.

### 4.1. Clinical and Preventive Implications

Given the high prevalence of TMD symptoms found among these young individuals, it is recommended that medical education institutions consider implementing screening and prevention programs. For example, workshops for students focused on raising awareness of parafunctional habits and stress management techniques may be beneficial. Additionally, students with significant symptoms should be encouraged to undergo specialist evaluations (by a dentist with expertise in orofacial pain or a maxillofacial surgeon), especially if the pain persists or worsens. Early interventions, such as intraoral protective devices (e.g., bruxism splints), physiotherapy exercises for the masticatory muscles, or psychological counseling, can help prevent the worsening and chronic progression of TMD.

Furthermore, universities could provide access to psychological counseling services, given that a significant percentage of students (approximately 16%) already present with moderate to severe anxiety symptoms, which not only affect their mental well-being but are also correlated with somatic problems such as TMD.

### 4.2. Study Limitations

Our study presents several limitations. First, the self-reported nature of data collection introduces a risk of both reporting and selection bias. Symptoms were subjectively assessed, without clinical confirmation, and participation was voluntary, which may have favored responses from students with existing concerns or symptoms. More broadly, voluntary response designs inherently involve bias, as each participant’s decision to engage is influenced by personal motivations and internal reasoning. These factors can affect not only who responds but also how symptoms are interpreted and reported.

Furthermore, the absence of a clinical examination according to DC/TMD criteria (e.g., muscle palpation, range of motion measurement, TMJ auscultation) limits the diagnostic accuracy of our findings. Without objective clinical evaluation, self-reported symptoms cannot be translated into formal TMD diagnoses. As such, the results should be interpreted as indicative of perceived symptom burden within a volunteer sample rather than as a precise prevalence estimate or a clinical case count.

Additionally, the study’s cross-sectional design precludes causal inference. While associations (e.g., between anxiety and pain) were identified, they cannot establish temporal or directional relationships. A longitudinal approach following students across academic years would offer valuable insights into whether anxiety precedes or results from TMD. The study also did not include a test–retest reliability assessment due to its anonymous and cross-sectional design. As responses could not be linked over time, we were unable to evaluate the stability of reported symptoms. Nonetheless, the instruments employed (DC/TMD axis I and II, PHQ-9, GAD-7, JFLS, OBC) are internationally validated and widely used in both clinical and research settings.

Future research may consider incorporating biological stress markers, such as salivary cortisol or alpha-amylase, to objectively validate psychosocial scores and strengthen correlations with TMD symptoms [14].

Furthermore, several potentially relevant variables were not assessed, including occlusal status, history of orofacial trauma, and prolonged computer use (which may contribute to cervical and masticatory strain). These omissions limit our ability to fully characterize risk profiles. We also did not collect data on participants’ general medical history or medication use, which could influence pain perception or reporting. These exclusions were intentional in order to preserve anonymity and maximize response rates. However, they reduce our capacity to control for confounding factors. Future studies should include structured medical history assessments to better account for systemic influences on orofacial symptoms.

Finally, the sample was drawn from a single institution with a medical academic background; expanding the study to other university centers (potentially within a national multicenter framework) could help determine whether the findings are generalizable or subject to local variation.

## 5. Conclusions

The present study revealed that temporomandibular joint (TMJ) disorders are common among students at the Faculty of Dental Medicine in Iași, although these disorders are generally of mild to moderate severity. Intermittent orofacial pain, joint noises, and episodes of transient mandibular locking were reported by a significant percentage of students, highlighting the need for future dental practitioners to be aware of this issue. A more pronounced impact of TMD was observed among female students, which aligns with general gender trends reported in the literature. Furthermore, the interdependence between TMD and psychosocial factors was confirmed: stress and anxiety appear to exacerbate TMD symptoms, likely through parafunctional habits and increased neuromuscular reactivity. This suggests that an effective and practical therapeutic approach should be interdisciplinary. By implementing preventive programs (such as stress management and reduction of parafunctional behaviors) and early intervention in symptomatic students, academic institutions can help reduce the burden of these disorders. In the future, additional studies are needed to investigate causal relationships further and evaluate the effectiveness of such preventive interventions among students.

Our findings may lay the groundwork for implementing strategies in the academic medical setting that enhance orofacial and overall health, ensuring the well-being of future dental professionals.

## Figures and Tables

**Table 1:** Key association analyses.

Association	Group 1	Group 2	χ^2^	df	*p*-Value
Orofacial pain vs. TMJ noises	Pain and noises: 79 (22.2%)	Neither: 132 (37.1%)	9.84	1	≈0.002
	Pain only: 76 (21.3%)	Noises only: 69 (19.4%)			
Temporal headache vs. orofacial pain	With headache: 131/155 (85%)	Without headache: 44/155 (29%)	72.3	1	<0.001
Nocturnal bruxism vs. orofacial pain	Bruxers: 58%	Non-bruxers: 38%	10.8	1	0.001
Nocturnal bruxism vs. morning jaw stiffness	Bruxers: 41%	Non-bruxers: 13%	32.7	1	<0.001
TMJ noises vs. oral behaviors	Noises present: mean 15.0	No noises: mean 10.6	*t*-test (approx.)	—	<0.01

df = degrees of freedom.

**Table 2:** Symptom prevalences by sex.

Symptom	Female % (*n* = 235)	Male % (*n* = 115)	χ^2^	df	*p*-Value
Orofacial pain	44.7% (105/235)	41.7% (48/115)	0.165	1	0.684
TMJ noises	43.0% (101/235)	39.1% (45/115)	0.325	1	0.568
Mandibular locking	20.4% (48/235)	19.1% (22/115)	0.020	1	0.887
Temporal headache	43.4% (102/235)	24.3% (28/115)	11.208	1	0.0008

df = degrees of freedom.

## Data Availability

The de-identified participant-level data that support the findings of this study are not publicly available due to privacy restrictions but are available from the corresponding author upon reasonable request and with permission of the Ethics Committee.

## Abbreviations

The following abbreviations are used in this manuscript:

TMD	Temporomandibular disorder
TMJ	Temporomandibular joint
DC/TMD	Diagnostic Criteria for Temporomandibular Disorders
GCPS	Graded Chronic Pain Scale
JFLS	Jaw Functional Limitation Scale
PHQ	Patient Health Questionnaire
GAD	Generalized anxiety disorder
OBC	Oral Behaviors Checklist
RDC/TMD	Research Diagnostic Criteria for Temporomandibular Disorders
CBT	Cognitive behavioral therapy
CT	Computed tomography
CBCT	Cone beam computed tomography
MRI	Magnetic resonance imaging
IQR	Interquartile range
